# ETHICAL IMPUTATION AND THE WILLINGNESS TO HELP PLOCS AMONG THE PUBLIC: THE MODERATING ROLE OF SOCIAL TRUST

**DOI:** 10.1093/geroni/igad104.2188

**Published:** 2023-12-21

## Abstract

In China, the population of older parents who lost their only child (PLOCs) is increasing and will keep growing for about 30 years. The PLOCs are thought to undertake the ethical imputation that they have not taken good care of the child, for the tradition emphasizes children as the center/fate of a family. This study aimed to investigate the public’s ethical imputation concerning PLOCs and its relationships to the willingness to help PLOCs. A nation-wide sample of 2029 (Mage = 36.62 years; SD = 10.06; 50.1% males) Chinses adults aged from 18 to 71 years provided demographic information and responded to the measures of ethical imputation, predicament awareness about PLOCs, social trust, and willingness to help PLOCs. We utilized Hayes’s (2018) PROCESS macro for SPSS (Model 1) to conduct analyses. The results indicated that the public had a relatively low level of predicament awareness about PLOCs (M = 4.28, SD = 2.09, range 1-12) and an above-average level of help willingness (M = 3.58, SD = 0.51, range 1-5). More importantly, the relationship between ethical imputation and help willingness was moderated by social trust. Specifically, ethical imputation negatively predicted help willingness among the public with higher levels of social trust; in contrast, this impact was not significant among the public with lower levels of social trust. These findings promote an understanding of how the public’s ethical imputation toward PLOCs and social trust can jointly influence their willingness to help PLOCs from the macro-system (i.e., the public attitude) perspective.

